# Two-year change in latent classes of comorbidity among high-risk Veterans in primary care: a brief report

**DOI:** 10.1186/s12913-022-08757-x

**Published:** 2022-11-12

**Authors:** Franya Hutchins, Joshua Thorpe, Xinhua Zhao, Hongwei Zhang, Ann-Marie Rosland

**Affiliations:** 1 VA Center for Health Equity Research and Promotion, VA Pittsburgh Healthcare System, 151C University Drive, Pittsburgh, PA 15240 USA; 2 Division of Pharmaceutical Outcomes & Policy, University of North Carolina Eshelman School of Pharmacy, Chapel Hill, NC, USA; 3 Department of Internal Medicine, University of Pittsburgh School of Medicine, Pittsburgh, PA, USA

**Keywords:** Multiple chronic conditions, Multimorbidity, Latent class analysis, Patient care management, Quality improvement

## Abstract

**Background:**

Segmentation models such as latent class analysis are an increasingly popular approach to inform group-tailored interventions for high-risk complex patients. Multiple studies have identified clinically meaningful high-risk segments, but few have evaluated change in groupings over time.

**Objectives:**

To describe population-level and individual change over time in latent comorbidity groups among Veterans at high-risk of hospitalization in the Veterans Health Administration (VA).

**Research design:**

Using a repeated cross-sectional design, we conducted a latent class analysis of chronic condition diagnoses. We compared latent class composition, patient high-risk status, and patient class assignment in 2018 to 2020.

**Subjects:**

Two cohorts of eligible patients were selected: those active in VA primary care and in the top decile of predicted one-year hospitalization risk in 2018 (*n* = 951,771) or 2020 (*n* = 978,771).

**Measures:**

Medical record data were observed from January 2016–December 2020. Latent classes were modeled using indicators for 26 chronic health conditions measured with a 2-year lookback period from study entry.

**Results:**

Five groups were identified in both years, labeled based on high prevalence conditions: Cardiometabolic (23% in 2018), Mental Health (18%), Substance Use Disorders (16%), Low Diagnosis (25%), and High Complexity (10%). The remaining 8% of 2018 patients were not assigned to a group due to low predicted probability. Condition prevalence overall and within groups was stable between years. However, among the 563,725 patients identified as high risk in both years, 40.8% (*n* = 230,185) had a different group assignment in 2018 versus 2020.

**Conclusions:**

In a repeated latent class analysis of nearly 1 million Veterans at high-risk for hospitalization, population-level groups were stable over two years, but individuals often moved between groups. Interventions tailored to latent groups need to account for change in patient status and group assignment over time.

**Supplementary Information:**

The online version contains supplementary material available at 10.1186/s12913-022-08757-x.

## Introduction

Identifying patients in primary care with the highest risk of poor outcomes, such as hospitalization and mortality, provides an opportunity for prevention. Although risk can be predicted with high levels of accuracy, ‘one size fits all’ intervention approaches have failed to reduce hospitalizations or improve health outcomes among the highest-risk patients. Instead, healthcare systems are increasingly looking to data-driven solutions to identify needs among the heterogeneous high-risk patient population.

Latent class analysis is a popular approach to empirically characterize subgroups within heterogenous high-risk patient populations [1–5]. These models can cluster high-risk patients based on their clinical profiles, allowing healthcare systems to design interventions tailored to each group’s utilization trends and outcomes [5–8]. While there are some common comorbidity patterns that appear among patients in multiple settings [9], it is becoming increasingly clear that the composition of empirically-derived clusters depend on the health system, setting, and data sources used [10].

A key gap in studies using latent class analysis in high-risk patient populations is determining whether and how latent comorbidity clusters (groups) change over time within the same setting and health system. Prior studies have shown that not all high-risk patients remain high risk over time [11, 12], and that individual-level comorbidity profiles evolve over time [13, 14]. If the latent groups themselves, or patient membership in a group, change over time, interventions tailored to latent patient groups will need to accommodate these expected changes in their design and evaluation.

We previously described latent groups among high-risk patients in the Veterans Health Administration (VA) based on comorbidity profiles in 2014 [15]. In this Brief Report we describe latent groups in this patient population in 2018 and 2020, evaluating change in group composition in the two years. We then examine how individuals moved between groups or out of the high-risk population over the time period. Our objective in this analysis was to describe the impact of population-level change over time in the VA high-risk patient population on latent comorbidity groups.

## Methods

### Patients

We conducted a repeated cross-sectional study comparing patients identified by their high-risk status in 2018 to those similarly identified in 2020. Patients were eligible in 2018 or 2020 if they were: 1) actively assigned to a VA primary care team during some portion of the year, and 2) in the top decile for risk of 1-year hospitalization based on the VA Care Assessment Needs (CAN) risk score during any week in the calendar year. The CAN risk score predicts the probability of the patient experiencing an inpatient admission to a VA hospital in the next 12 months The score is generated weekly for all active VA primary care patients with inputs that include demographic, utilization, lab, diagnoses, and medication data [16]. The top decile cut-off was chosen as it is widely used throughout the VA to determine eligibility for initiatives targeting patients with complex health care needs. Each patient’s study entry date was defined as the first day in the observed year with a top decile CAN score recorded. This work was designated non-research, and requirement for IRB approval waived, since it was carried out as a quality improvement evaluation under the terms of a signed attestation of non-research from the VHA Office of Primary Care (OPC).

### Data sources

Patient sociodemographic data, hospitalization dates, and outpatient utilization were obtained from the VA administrative Corporate Data Warehouse (CDW). Mortality data were obtained from VA Vital Status files, which combine information from the VA, Medicare, and Social Security. Primary care site was classified as hospital or stand-alone outpatient clinic using standard VA definitions.

Inputs for latent class models were indicators for 26 health conditions, chosen based on the U.S. Department of Health and Human Services’ multiple chronic conditions framework [17, 18]. Conditions specifically relevant to Veteran health, such as PTSD, were added, and conditions without enough sample prevalence to be included in latent class models, such as HIV and autism, were excluded. Using a 2-year lookback period from cohort entry, each chronic condition was defined as present if the patient had one outpatient or inpatient care encounter containing an ICD-10 code for that condition.

### Statistical analysis

Comorbidity groups were modeled using latent class analysis (LCA) [19]. LCA is a probabilistic clustering approach that assumes patterns in response variables (e.g., medical conditions) reflect meaningful, discrete latent groups of individuals. Estimates from LCA include (1) the identification of latent classes (groups) based on patterns of comorbidity, and (2) a predicted probability of group membership for each patient per group. The predicted probability provides a measure of uncertainty for patient class assignments.

Models of 2 to 7 classes were tested among patients observed in 2018 with the final model chosen based on a combination of clinical interpretability and model fit statistics including AIC, BIC, adjusted BIC, and entropy. Patients were assigned to a latent group if their predicted probability of membership was ≥50%. If no probability was ≥50%, patients were designated as ‘unassigned,’ allowing us to account for uncertainty in class membership. This process was then repeated for patients observed in 2020.

We chose this repeated cross-sectional design as opposed to formally testing longitudinal class status with latent transition analysis [19] to represent the likely application of these models in practice. Specifically, selecting high-risk patients in 2018 and separately in 2020 allows for patients to move in and out of the high-risk population, and reflects the repeated cross-sectional nature of data from primary care encounters.

To assess the population-level stability of latent groups, we compared the prevalence of chronic conditions by latent group for patients observed in 2018 to those in 2020. To describe individual-level changes over time, we followed the 2018 patients through calendar year 2020. Patients were then categorized by their status in 2020 as 1) maintaining a top decile (e.g. “high-risk”) CAN score, 2) moving to a lower-risk CAN score, 3) death, or 4) no longer active in VA primary care. We also examined whether and how individuals’ latent group assignments changed between the two years.

### Supplemental analyses

We completed a supplemental descriptive comparison of patients who were assigned to the same group in both years to those who were assigned to different groups. See Supplemental Digital Content for additional methods.

### Software

Latent class analysis models were run in MPLUS version 8.2 (Los Angeles, CA: Muthén & Muthén). All other analyses were run in STATA 14 (College Station, TX: StataCorp LP).

## Results

### Patient characteristics

We identified 951,771 high-risk patients in 2018 and 978,771 in 2020. Patient characteristics were similar in the two years (Table 1). Mean age was 66.1 (standard deviation = 13.1) and 66.6 (13.2) in 2018 and 2020 respectively. Most patients (92 and 91%) were male. Most patients identified as non-Hispanic White (64.2 and 63.0%) or non-Hispanic Black (22.7 and 23.4%). Over 25% had indicators of low income in both years.

**Table 1:** High-Risk Primary Care Patient Characteristics

	2018 Patients *N* = 951,771	2020 Patients *N* = 978,771
**Sociodemographic Characteristics**		
Male, %	92.0	91.2
Age in years, mean (sd)	66.1 (13.1)	66.6 (13.2)
Race/Ethnicity, %		
Non-Hispanic Black	22.7	23.4
Non-Hispanic White	64.2	63.0
Hispanic	6.2	6.4
Non-Hispanic Other	3.5	3.6
Missing	3.5	3.6
Low income, %^a^	30.3	26.6
Housing instability, %^a^	19.0	17.9
Currently married, % ^b^	39.7	40.5
Rural residence (versus urban), % ^b^	30.7	29.3
**Health Characteristics**		
Number of chronic conditions (range 0–26), mean (sd)^a^	6.9 (2.6)	7.1 (2.7)
Gagne comorbidity index, mean (sd)^a^	3.8 (2.9)	3.3 (2.7)
CAN risk score (predicted probability of 1-year hospitalization, range 0–1), mean (sd)	0.33 (0.13)	0.32 (0.13)
Receives VA primary care at hospital-based (versus community-based) clinic, % ^b^	54.1	53.3
**Health Outcomes in the 12 Months Following Study Entry Date** ^c^		
Mortality, %	8.4	9.1
Any acute hospitalization, %	24.0	20.0

*Abbreviations: CAN* Care Assessment Needs, *VA* Veterans Health Administration

^a^Data for low-income status, housing instability, number of chronic condition diagnoses, and Gagne score are collected with a 24-month lookback period from cohort entry date

^b^The following variables contained some missing data. Marital status and primary care location, < 1%; Rural residence, 1.3% (2018) and 3.9% (2020)

^c^Data for mortality and hospitalization are collected prospectively for 12 months following individuals’ cohort entry date in each year. These data were available through March 2021. *n* = 359 patients in the 2020 cohort are excluded from the mortality and hospitalization values due to having < 12 months of prospective data available (*n* = 978,412)

### Latent class analysis

**Fig. 1:**
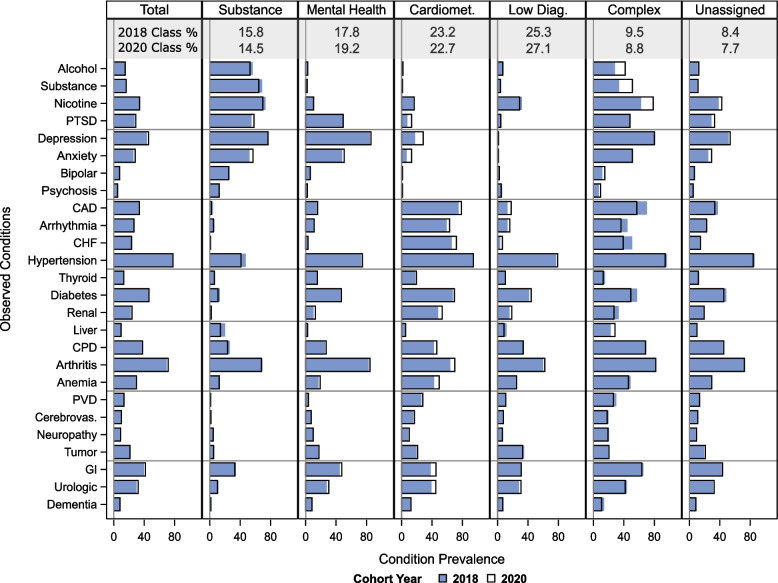
Stability in 2018 versus 2020 Prevalence of Chronic Conditions, by High-Risk Patient Latent Groups (2018 *n* = 951,771; 2020 *n* = 978,771). Abbreviations: Cardiomet.; Cardiometabolic; Low Diag., Low Diagnoses; Alcohol, Alcohol Use Disorder; Substance, Substance Use Disorder; Nicotine, Nicotine Use Disorder; PTSD, Post-Traumatic Stress Disorder; CAD, Coronary Artery Disease; CHF, Congestive Heart Failure; Thyroid, Thyroid Disorders; Renal, Chronic Renal Failure; Liver, Chronic Liver Disease; CPD, Chronic Pulmonary Disease; PVD, Peripheral Vascular Disease; Cerebrovas., Cerebrovascular Disease; Tumor, Malignant Tumor; GI, Gastrointestinal Disorders. Data are observed prevalence of chronic condition diagnoses among the 2018 (blue bar) and 2020 (black outline) panels. Patients are assigned to a latent group if the predicted probability of group membership is ≥50%, otherwise categorized as “unassigned”

In latent class models testing 2 to 7 groups, each additional group improved BIC but worsened entropy (see Table, Supplemental Digital Content 1). A 5-group model was chosen as the best balance between model fit and clinical interpretability. Similar results were found for 2020 LCA models. In 2018, 871,613 (91.6%) of patients, and in 2020 903,041 (92.3%), were assigned to a group using the cut-off value of 50% predicted probability. We labeled groups based on the high-prevalence conditions within each, relative to the overall average. Profiles of chronic conditions within groups were similar in both years (see Fig. 1). We observed a “Mental Health” group, with high prevalence of post-traumatic stress disorder, depression, and anxiety, a “Substance Use Disorders” group, with high prevalence of mental health diagnoses as well as alcohol, substance, and nicotine use disorders, a “Cardiometabolic” group, with high prevalence of coronary artery disease, congestive heart failure, and arrhythmia, a “Low Diagnosis” group, with low prevalence of most conditions, and a “High Complexity” group, with high prevalence of conditions across multiple physiologic systems. Patients assigned to the High Complexity group had an average of 11.3 (standard deviation = 2.1) chronic disease diagnoses in 2018, compared to the overall average of 6.9 (2.6). See Table, Supplemental Digital Content 2 for patient characteristics by latent group.

### Population-level stability

Between 2018 and 2020, no condition changed in its overall cohort prevalence by more than 2.5 percentage points (see Table, Supplemental Digital Content 3 for prevalence). Within groups, condition prevalence was also generally stable (Fig. 1). Exceptions include shifts in mental health, substance use disorder, and cardiovascular conditions, especially within the Cardiometabolic and High Complexity groups. For example, diagnoses of alcohol use disorder increased from 28 to 42% among those assigned to the High Complexity group, while depression diagnoses increased from 18 to 29% among those assigned to the Cardiometabolic group.

### Individual-level stability

**Fig. 2:**
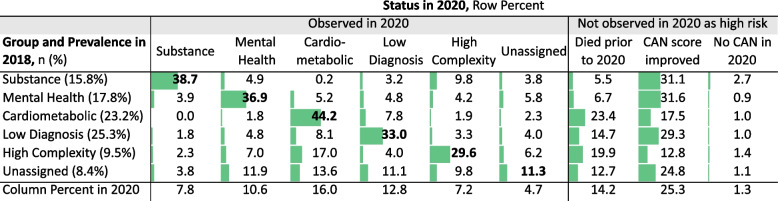
Individual Movement in Latent Group Assignment or Status among High-Risk Patients in 2018 to 2020 (n = 951,771). Abbreviations: CAN, Care Assessment Needs. Data are row percent, representing patient status in 2020 by latent group assignment in 2018. All patients observed in 2018 are included. Patients are assigned to a latent group if the predicted probability of group membership is ≥50%, otherwise categorized as “unassigned”

Among the 951,771 patients observed in 2018, 563,725 (59.2%) were also observed to have a high-risk CAN score in 2020. The remaining patients either died prior to 2020 (14.2%), had an improved CAN score and so were no longer designated as high risk (25.3%), or did not engage in primary care through the VA and therefore had no CAN score recorded in 2020 (1.3%, see Fig. 2). The High Complexity group was the least likely to be observed with an improved CAN score in 2020 (12.8%). In contrast, nearly one third of patients assigned in 2018 to the Substance, Mental Health, and Low Diagnosis groups were observed with an improved CAN score in 2020 and no longer qualified as high-risk by our definition.

Of the 563,725 patients with a high CAN score in both 2018 and 2020, 40.8% (*n* = 230,185) had a different group assignment in each year (see Table, Supplemental Digital Content 4). Patients assigned to the Cardiometabolic group in 2018 were the most likely to be observed and assigned to the same group in 2020 (76%). Patients assigned to the Substance Use Disorders group and those unassigned in 2018 were the most likely to move into the High Complexity group in 2020 (16% of each). Most high-CAN patients who did not match with a group (‘unassigned’) in 2018 were matched with a specific group in 2020 (82%).

### Supplementary analyses

Our descriptive comparison of patients assigned to the same versus different groups in the two years found few meaningful differences, with similar average age, income level, and number of chronic condition diagnoses (see Table, Supplemental Digital Content 5 for patient characteristics).

## Discussion

Latent class analyses in 2018 and 2020 identified a similar set of five groups based on profiles of chronic conditions among Veterans at high-risk for hospitalization. The prevalence of conditions within groups was stable over the two years. However, on the individual level, many patients moved out of the target population or moved to a different group assignment in that time. Over 25% of patients labeled high risk in 2018 had an improved risk score in 2020, and 24% remained high risk but were assigned to a different latent group.

The groups we identified were similar to those reported in previous cohorts of high-risk VA patients, including mental health, substance-associated, and cardiometabolic groups [3, 20]. The categories of cardiovascular, metabolic, and psychiatric groups have also been reported in segmentation studies of high-risk patient populations in other systems and settings [1, 4, 10].

Few studies have examined changes in latent comorbidity groups in the same population over time. Violán et al. described comorbidity clusters among over 400,000 adult primary care patients with multimorbidity in Catalonia, Spain [21]. Their analysis found 10 groups including a large “nonspecific” group (42%). In contrast to our analysis, most patients were assigned to the same category five years later. In a smaller (*n* = 2931) prospective cohort of adults age 60 and over with multimorbidity in Sweden, Vetrano et al. used a clustering algorithm to identify six patient groups based on comorbidity profiles [14]. In this case, clustering repeated at 6 and 12 years of follow-up showed substantial movement between clusters over time.

Our findings have important implications for the design and evaluation of clinical interventions tailored to patient segments. Segmentation models are increasingly seen as a promising way to identify common subgroups among complex patients [10, 22]. Establishing the validity and stability of comorbidity clusters, from both the population- and patient-level, is fundamental to intervention design. Our work shows that group-tailored interventions may need to be planned as adaptable or limited-term to stay relevant to shifting patient clinical profiles. In addition, the choice of evaluation metrics and approach for tracking long-term outcomes by group should account for naturally occurring changes in patient risk status and clinical profiles over time. It is not clear whether changes in latent class profiles were due to movement of patients in and out of high-risk status, or the accumulation of chronic disease burden on patients observed in both time frames. For applications where the goal is to understand why chronic disease profiles change for individuals, additional analyses would be needed to better understand the drivers of change in comorbidity profiles over time.

The strengths of this study include its ability to leverage data representing a large sample of high-risk Veterans receiving care across the US in an integrated healthcare system. In addition, the timeframe of data available allowed us to compare patients over time with two-year lookback periods for chronic condition diagnoses at each timepoint. A limitation to consider is the generalizability of the Veteran population to other patient populations. Women in particular are underrepresented compared to the general population [23]. Finally, some patients may have received care outside the VA, meaning diagnoses may be missed in our data.

In the national population of Veterans in VA primary care at high risk for hospitalization, latent class analysis identified informative patient groups based on chronic condition profiles that could be used to inform group-tailored interventions. Population-level group profiles remained stable over two years, but individual risk levels and group assignments showed meaningful change. It is critical that healthcare systems evaluate population- and individual-level stability of segmentation models prior to incorporating these patient profiles into care or intervention design.

## Supplementary Information

**Additional file 1.** Supplemental Digital Content

## Data Availability

The dataset and code supporting the conclusions of this article can be requested from the corresponding author but can only be made available after the completion of all government requirements such as the HIPAA deidentification standard, related VHA regulations and directives, and the Medicare and Medicaid Analysis Center’s Rules of Behavior.
