# Supplementary material for: Two-year change in latent classes of comorbidity among high-risk Veterans in primary care: a brief report
Source: BMC Health Serv Res. 2022 Nov 12;22:1341. doi: 10.1186/s12913-022-08757-x (PMC9652993; doi:10.1186/s12913-022-08757-x)
Supplement: Supplementary file 1 — Additional file 1. Supplemental Digital Content [file 12913_2022_8757_MOESM1_ESM.docx]

SUPPLEMENTAL MATERIALS

Two-Year Change in Latent Classes of Comorbidity among High-Risk Veterans in Primary Care: A Brief Report

**Table of Contents**

| Content | Page |
| --- | --- |
| eTable 1. Goodness of Fit Comparisons of LCA Models with Different Number of Groups, 2018 Patients (n=951,771) | 2 |
| eTable 2. Patient Characteristics by Latent Group Assignment, 2018 High-Risk Primary Care Patients (n=951,771) | 3 |
| eTable 3. Overall Prevalence of Chronic Conditions, High-Risk Patients in 2018 and 2020 | 4 |
| eTable 4. Latent Group Assignment in 2018 and 2020, Among Patients Observed in Both Years (n=563,725) | 5 |
| Supplemental Analysis: Descriptive Comparison of Patients Assigned to the Same versus Different Latent Groups (2018 and 2020) | 6 |
| eTable 5. Patient Characteristics, Among Primary Care Patients Observed as High-Risk in 2018 and 2020 (n=563,725) | 6 |

**eTable 1.** Goodness of Fit Comparisons of LCA Models with Different Number of Groups, 2018 Patients (n=951,771)

| Number Groups | df | loglikelihood | AIC | BIC | Sample-size adjusted BIC | entropy |
| --- | --- | --- | --- | --- | --- | --- |
| 2 | 53 | -11917714.87 | 23835535.74 | 23836159.34 | 23835990.9 | 0.701 |
| 3 | 80 | -11799789.06 | 23599738.12 | 23600679.41 | 23600425.16 | 0.651 |
| 4 | 107 | -11729011.99 | 23458237.98 | 23459496.95 | 23459156.89 | 0.658 |
| 5 | 134 | -11682781.8 | 23365831.6 | 23367408.25 | 23366982.39 | 0.647 |
| 6 | 161 | -11653788.71 | 23307899.42 | 23309793.75 | 23309282.09 | 0.628 |
| 7 | 188 | -11627720.26 | 23255816.52 | 23258028.54 | 23257431.07 | 0.627 |

**eTable 2.** Patient Characteristics by Latent Group Assignment, 2018 High-Risk Primary Care Patients (n=951,771)

|  | Total | Substance | Mental Health | Cardio-metabolic | High Complexity | Low Diagnosis | Unassigned |
| --- | --- | --- | --- | --- | --- | --- | --- |
| Total N | 951,771 | 150,183 | 169,040 | 220,962 | 90,852 | 240,576 | 80,158 |
| Total % | 100 | 15.8 | 17.8 | 23.2 | 9.5 | 25.3 | 8.4 |
| Sociodemographic Characteristics | | | | | | | |
| Male, % | 92 | 87.1 | 82.3 | 97.9 | 95.6 | 94.6 | 93.1 |
| Age in years, mean (sd) | 66.1 (13.1) | 52.4 (12.9) | 62.7 (12.7) | 74.3 (9.9) | 67.2 (8.6) | 68.7 (10.5) | 67.2 (10.6) |
| Race/Ethnicity, % |  |  |  |  |  |  |  |
| Non-Hispanic Black | 22.7 | 28.8 | 22.1 | 17.2 | 22.4 | 24.8 | 21.4 |
| Non-Hispanic White | 64.2 | 57.2 | 62.3 | 70.7 | 66.1 | 62.6 | 65.7 |
| Hispanic | 6.2 | 6.8 | 8.3 | 5.1 | 4.6 | 5.8 | 6.0 |
| Non-Hispanic Other | 3.5 | 4.3 | 3.8 | 3.2 | 3.5 | 3.1 | 3.5 |
| Missing | 3.5 | 2.9 | 3.4 | 3.8 | 3.3 | 3.7 | 3.5 |
| Census tract socioeconomic status index quintiles, % | | | | | | | |
| 1 (lowest) | 29.3 | 34.4 | 27.0 | 25.3 | 30.5 | 31.1 | 29.0 |
| 2 | 21.8 | 20.9 | 21.9 | 22.0 | 22.8 | 21.6 | 22.2 |
| 3 | 18.5 | 16.8 | 18.9 | 19.8 | 18.6 | 18.0 | 18.9 |
| 4 | 16.1 | 14.6 | 16.9 | 17.2 | 15.6 | 15.4 | 16.1 |
| 5 (highest) | 13.6 | 11.8 | 14.9 | 15.0 | 12.1 | 13.0 | 13.3 |
| Missing | 0.7 | 1.5 | 0.4 | 0.6 | 0.4 | 0.8 | 0.5 |
| Low income, % | 30.3 | 33.1 | 19.0 | 32.3 | 28.5 | 35.9 | 28.5 |
| Housing instability, % | 19.0 | 50.6 | 15.3 | 7.3 | 25.1 | 11.4 | 15.6 |
| Currently married, % ƚ | 39.7 | 21.8 | 43.0 | 51.1 | 42.0 | 36.5 | 41.8 |
| Health and Healthcare Characteristics | | | | | | | |
| Number of comorbidities, mean (sd) | 6.9 (2.6) | 7.0 (2.1) | 6.2 (1.8) | 7.9 (2.1) | 11.3 (2.1) | 4.9 (1.7) | 7.1 (1.9) |
| CAN score ≥ 95th pctl versus 90-94, % | 33.9 | 37.3 | 20.2 | 43.0 | 65.3 | 21.7 | 31.6 |
| VA primary care at hospital-based clinic (versus stand-alone outpatient clinic), % | 54.1 | 57.5 | 53.8 | 51.8 | 55.0 | 54.4 | 53.2 |
| Outcomes | Total | Substance | Mental Health | Cardio-metabolic | High Complexity | Low Diagnosis | Not Assigned |
| One-year mortality, % | 8.4 | 3.1 | 3.9 | 13.8 | 11.4 | 9.1 | 7.4 |
| Any hospitalization, % | 24.0 | 25.1 | 17.3 | 27.6 | 33.7 | 21.7 | 21.8 |

Abbreviations: CAN, Care Assessment Needs; pctl, percentile; VA, Veterans Health Administration.

ƚ Marital status contained 0.6% missing data in 2018.

**eTable 3.** Overall Prevalence of Chronic Conditions, High-Risk Patients in 2018 and 2020

| Condition, % | 2018 | 2020 |
| --- | --- | --- |
| Alcohol Use Disorder | 15.3 | 15.1 |
| Substance Use Disorder | 16.4 | 16.4 |
| Nicotine Use | 34.4 | 33.9 |
| Post-Traumatic Stress Disorder | 27.3 | 28.9 |
| Depression | 43.8 | 45.6 |
| Anxiety | 25.7 | 28.0 |
| Bipolar Disorder | 7.6 | 7.5 |
| Psychosis | 5.2 | 4.9 |
| Coronary Artery Disease | 33.8 | 33.6 |
| Cardiac Arrhythmias | 26.2 | 26.5 |
| Congestive Heart Failure | 22.7 | 23.4 |
| Hypertension | 78.1 | 78.0 |
| Thyroid | 12.6 | 13.1 |
| Diabetes | 45.9 | 46.2 |
| Chronic Renal Failure | 22.1 | 23.8 |
| Chronic Liver Disease | 10.7 | 9.3 |
| Chronic Pulmonary Disease | 37.6 | 37.7 |
| Arthritis | 69.4 | 71.5 |
| Anemia | 28.2 | 29.8 |
| Peripheral Vascular Disease | 13.4 | 13.4 |
| Cerebrovascular Disease | 9.7 | 10.0 |
| Neuropathy | 8.5 | 8.9 |
| Malignant Tumor | 20.6 | 21.2 |
| Gastrointestinal Disorders | 39.7 | 41.6 |
| Urologic | 29.7 | 32.0 |
| Dementia | 8.5 | 8.1 |

**eTable 4.** Latent Group Assignment in 2018 and 2020 (Row Percent), Among Patients Observed in Both Years (n=563,725) ƚ

|  | **Group in 2020, n (Row Percent)** | | | | | |  |
| --- | --- | --- | --- | --- | --- | --- | --- |
| **Group in 2018** | Substance | Mental Health | Cardio-metabolic | Low Diagnoses | High Complexity | Unassigned | Total |
| Substance | 63.8 | 8.1 | 0.3 | 5.3 | 16.1 | 6.3 | 91,211 |
| Mental Health | 6.5 | 60.6 | 8.6 | 7.9 | 6.9 | 9.5 | 102,763 |
| Cardiometabolic | 0.0 | 3.1 | 76.2 | 13.4 | 3.2 | 4.0 | 128,291 |
| Low Diagnoses | 3.3 | 8.7 | 14.7 | 60.0 | 6.0 | 7.2 | 132,310 |
| High Complexity | 3.4 | 10.5 | 25.8 | 6.0 | 44.9 | 9.4 | 59,883 |
| Unassigned | 6.2 | 19.3 | 22.0 | 18.1 | 15.9 | 18.4 | 49,267 |
| Total | 74,415 | 101,067 | 152,573 | 122,118 | 68,597 | 44,955 | 563,725 |

ƚ Data are number and row percent, representing patient latent group in 2020 by latent group in 2018. Patients are assigned to a latent group if the predicted probability of group membership is ≥ 50%, otherwise categorized as “unassigned.”

**Supplemental Analysis: Descriptive Comparison of Patients Assigned to the Same versus Different Latent Groups (2018 and 2020)**

eTable 5 shows patient characteristics for those who were observed as high-risk in both 2018 and 2020 (n=563,725), comparing patients who were assigned to the same group in both years to those who were assigned to different groups.

Outpatient utilization variables including primary care, mental health, and emergency, and inpatient care were drawn from VA encounter data using relevant stop codes. Data for low-income status, housing instability, number of chronic condition diagnoses, and Gagne score are collected with a 24-month lookback period from 2018 cohort entry date. Data for VA Healthcare Utilization are collected prospectively for 12 months following the 2018 cohort entry date. The following variables contained some missing data. Marital status and primary care location, <1%; Rural residence, 1.2%.

**eTable 5.** Patient Characteristics, Among Primary Care Patients Observed as High-Risk in 2018 and 2020 (n=563,725)

|  | All patients at high-risk in both 2018 and 2020 | Patients assigned to the same latent class | Patients assigned to a different latent class |
| --- | --- | --- | --- |
|  | N=563,725 | n=333,540 (59.2%) | n=230,185 (40.8%) |
| **Sociodemographic Characteristics** |  |  |  |
| Male, % | 91.5 | 91.0 | 92.2 |
| Age in years, mean (sd) | 65.6 (12.1) | 65.3 (13.0) | 66.1 (10.6) |
| Race/Ethnicity, % |  |  |  |
| Non-Hispanic Black | 24.7 | 24.3 | 25.1 |
| Non-Hispanic White | 62.4 | 62.6 | 62.2 |
| Hispanic | 6.2 | 6.4 | 6.0 |
| Non-Hispanic Other | 3.5 | 3.5 | 3.5 |
| Missing | 3.2 | 3.2 | 3.3 |
| Low income, %* | 29.9 | 30.1 | 29.7 |
| Housing instability, %* | 20.3 | 20.7 | 19.7 |
| Currently married, % ƚ | 38.2 | 38.1 | 38.2 |
| Rural residence (versus urban), % ƚ | 29.2 | 29.2 | 29.3 |
| **Health Characteristics** |  |  |  |
| Number of chronic conditions (range 0-26), mean (sd)* | 7.1 (2.6) | 7.0 (2.6) | 7.2 (2.6) |
| Gagne comorbidity index, mean (sd)* | 3.8 (2.7) | 3.8 (2.7) | 3.7 (2.7) |
| CAN risk score (predicted probability of 1-year hospitalization, range 0-1), mean (sd) | 0.3 (0.1) | 0.3 (0.1) | 0.3 (0.1) |
| Receives VA primary care at hospital-based (versus community-based) clinic, % ƚ | 56.9 | 56.8 | 57.1 |
| **VA Healthcare Utilization in the 12 Months Following Study Entry Date** ǂ | | | |
| Number of in-person primary care encounters, mean (sd) | 5.2 (4.5) | 5.1 (4.4) | 5.4 (4.6) |
| Any mental health clinic in-person encounters, % | 52.8 | 50.7 | 55.8 |
| Any mental health clinic phone encounters, % | 22.6 | 22.6 | 22.6 |
| Number emergency/urgent care encounters, mean (sd) | 1.8 (2.6) | 1.7 (2.6) | 1.8 (2.7) |
| Any palliative care and hospice outpatient encounters % | 2.0 | 1.9 | 2.1 |
| Any acute hospitalization, % | 27.3 | 26.8 | 28.0 |

Abbreviations: CAN, Care Assessment Needs; VA, Veterans Health Administration.
